# Correlation between stress, stress-coping and current sleep bruxism

**DOI:** 10.1186/1746-160X-6-2

**Published:** 2010-03-05

**Authors:** Maria Giraki, Christine Schneider, Ralf Schäfer, Preeti Singh, Matthias Franz, Wolfgang HM Raab, Michelle A Ommerborn

**Affiliations:** 1 Department of Operative and Preventive Dentistry and Endodontics, Heinrich-Heine-University Düsseldorf, Moorenstr 5, 40225 Düsseldorf, Germany; 2 Clinical Institute of Psychosomatic Medicine and Psychotherapy, Heinrich-Heine-University Düsseldorf, Moorenstr 5, 40225 Düsseldorf, Germany

## Abstract

**Background:**

Stress is discussed as a potential factor in the development of sleep bruxism (SB). The aim of this study was to investigate whether specific stress-factors correlate with SB-activity.

**Methods:**

Sixty-nine subjects, of which 48 were SB-patients, completed three German questionnaires assessing different stress-parameters and stress-coping-strategies: Short questionnaire for recognition of stress-factors (Kurzer Fragebogen zur Erfassung von Belastungen, KFB), Questionnaire for recuperation and strain (Erholungs-Belastungs-Fragebogen, EBF-24 A/3) and the stress-coping questionnaire (Stressverarbeitungsfragebogen-78, SVF-78). The diagnosis of SB was based on the clinical criteria of the American Academy of Sleep Medicine (AASM). The degree of SB-activity was measured by the Bruxcore-Bruxism-Monitoring-Device (BBMD, Bruxcore, Boston, USA), worn for five consecutive nights and analyzed using a computer-based method. Non-parametric Spearman correlation coefficients, rho, were calculated between the psychometric data and the amount of SB-activity measured by a pixel score of the BBMD.

**Results:**

Significant correlations were found for 'daily problems' (r = 0.461, p < 0.01), 'trouble at work' (r = 0.293), 'fatigue' (r = 0.288), 'physical problems' (r = 0.288) and the coping-strategy 'escape' (r = 0.295) (all p < 0.05).

**Conclusions:**

Within the limitations of this study it could be shown that subjects with high SB-activity tend to feel more stressed at work and in their daily life, which in turn might influence their physical state. These subjects also seem to deal with stress in a negative way. However, due to the rather low to almost moderate correlation coefficients and the descriptive character of the study, further investigations are necessary to examine a possible causal relationship.

## Background

Sleep bruxism (SB) is defined as a 'stereotyped movement disorder characterized by grinding or clenching of the teeth during sleep usually associated with sleep arousal' [1]. It might lead to abrasive tooth wear, hypermobility of teeth, tooth hypersensitivity, hypertrophy of the masticatory muscles and pain in the masticatory muscles [2]. There are no gender differences in SB. SB can also be found in children [3,4], but the age distribution reveals a larger incidence in individuals between 20 to 45 years of age [1,5].

Presently, the etiology of SB is not well defined. Different etiological factors have been investigated, e.g. occlusal interferences [6,7], transient sleep arousal episodes [8-10], a side imbalance in striatal D2 receptor binding [11,12], personality traits [5,13-16], psychosocial factors [17-19] and psychological stress [17,18,20-22]. At the same time, the multifactorial nature of SB is widely accepted [5,12,23-27].

Several authors have investigated stress as one of the causal agents of SB. In 1975 *Rugh and Solberg *reported that SB seemed to appear after days which were exhausting and stressful [28]. In an epidemiological study on British, German and Italian population samples, self-reported SB was also positively associated with a highly stressful lifestyle [21]. In another research on 1339 employees of a Finnish broadcasting company, frequent bruxism was significantly associated with severely stressful situations at work. Furthermore, frequent bruxism was significantly positively associated with the number of occupational health care and dental visits. It was concluded that bruxism may reveal ongoing stress in normal work life [22]. In a follow-up study of 30-50 year-old employees (n = 211) of the Finnish broadcasting company it could be reconfirmed that psychosocial factors and perceived stress should not be ignored [17]. In the same study it could also be shown that smoking was significantly positively associated with frequent bruxism. It was concluded that tobacco use may both amplify the patient's pain response and provoke bruxism [17]. Another epidemiological study examined the relationship between psychosocial job stress and SB in a Japanese population of 1944 male and 736 female factory workers. The study found that SB was weakly associated with some aspects of job stress in men among the Japanese working population [18].

Two further studies demonstrated an association between SB and an overtly ambitious character or behavior (Type A), which in turn is related to a stressful life [14,15]. A psychometric study found a significantly higher stress perception in bruxers compared to healthy controls [29]. Studies on urinary catecholamines in bruxers, indicating stressful states, detected a significant association of urinary epinephrine and dopamine with bruxism in children [30], as well as a positive relationship between increased urinary epinephrine and high levels of sleep masseter muscle activity [31]. Animal experiments with rats concerning the relationship between emotional stress and brux-like activity of their masseter muscles have also suggested a positive correlation [32].

Furthermore *Schneider et al. *investigated stress-coping strategies in patients with SB compared to non-bruxing controls. They observed a significant difference in positive coping strategies, which are capable of reducing stress, between the two groups. SB-patients reported significantly less positive coping strategies, like 'reaction control' and 'positive self-instructions'. Based on the above, a deficit of functional coping strategies in SB-patients could be demonstrated [33].

In contrast to these findings, two other studies showed that there was no relationship between the degree of SB and self-reported stress [34,35]. Another study, found no significant differences between bruxers and controls with respect to perceived stress during the previous year [36].

Overall, the majority of studies suggest an association between stress and SB, although increased SB as a direct consequence of diurnal stress could not be proved. It remains debatable as to which specific stress-factors correlate with SB.

According to the transactional model of stress and coping [37], stress depends on the impact of an external stressor, which is mediated by the appraisal of the stressor (primary appraisal) on the one hand, and the appraisal of the individual's capabilities to handle the situation (secondary appraisal) on the other hand. Coping represents the actual strategy of an individual to deal with the stressor. Dispositional coping styles are generalized ways of behaving in stressful situations, stable across time and circumstances. However, investigations regarding the correlation between stress-coping and SB are rare.

Therefore, the aim of the present study was to investigate whether specific stress related factors and coping strategies, from different areas of life, correlate with SB. A reliable and concurrently practicable instrument should be used, in order to measure the degree of SB. This allows for a high number of participants to be easily examined.

The literature states numerous methods with different validity and practicability for the assessment of SB-activity: laboratory polysomnographic recordings as the gold standard [9,10,12,27,38], portable electromyographic (EMG) recordings [28,31,39], accelerometer systems [40], a force-based bruxism detection system [41], questionnaires for self-evaluation of bruxism [14,21,22], dental examinations [30,42], examination of stone casts [43] as well as measuring abrasion on a diagnostic plate, the Bruxcore Bruxism-Monitoring Device (BBMD, Bruxcore, USA) [44]. Recently, it has been shown that the BBMD, in combination with a newly computer-based analyzing-method, is a reliable and feasible instrument to quantify abrasion as an indication for current SB [45].

The hypothesis used in this study was that high levels of specific stress related factors are associated with a high level of SB-activity. Additionally, it was hypothesized that handling stress in a non-effective way may lead to high SB-activity.

## Methods

### Sample

The sample consisted of 69 subjects, of which 48 were SB-patients. They were all German native speakers and responded to announcements in local newspapers and placards on campus. Subjects were screened following a thorough dental functional analysis [recommendations of the German Society of Dentistry and Oral Medicine (DGZMK)]. The diagnosis of SB was made clinically and was based on the criteria of the American Academy of Sleep Medicine (AASM) [1]. Individuals that met the following criteria were included in the study: healthy adults, aged between 20 to 40 years and sleeping partner reports of grinding sounds during the night in the last 6 months. Additionally, at least one of the following symptoms: self report of muscle fatigue or pain on awakening, abnormal tooth wear or shiny spots on restorations and masseter hypertrophy upon digital palpation. Exclusion criteria were: current dental treatment, cognition of SB for more than ten years, severe psychological disorders and/or the use of antipsychotic psychotropic drugs, alcohol-abuse, central nervous system and/or peripheral nervous system disorders, more than two missing molars (excluding third molars), the presence of a prosthesis or extensive prosthetic restorations and the presence of gross malocclusion. Healthy adults, from whom SB could be excluded, represented the non-bruxing subjects. Exclusion criteria were the same as for the SB-patients, as well as any signs and symptoms of SB. SB-patients did not differ from the non-bruxing subjects with respect to age, gender, education and the type of occlusal guidance scheme. The sociodemographic characteristics are displayed in Table 1.

**Table 1:** Sociodemographic characteristics and data related to the type of occlusal guidance scheme

	SB-patients (n = 48)	Non-bruxing subjects (n = 21)	*P*
Age	29.38 (± 4.41)	28.10 (± 5.75)	n.s.^a^
Gender	33 F, 15 M	13 F, 8 M	n.s.^b^
Education^c^	2 x_1_; 29 x_2_; 17 x_3_	1 x_1_; 16 x_2_; 4 x_3_	n.s.^b^

Canine protected articulation (%)	2.1	9.5	n.s.^b^
Anterior protected articulation (%)	25.0	28.6	n.s.^b^
Group function (%)	72.9	61.9	n.s.^b^

n.s. = not significant; * = p < 0.05; ** = p < 0.01; *** = p < 0.001

^a^*t*-test; values are presented as mean ± standard deviation

^b^Chi-square test

^c^Education was divided into three grades: x_1_-10 yr of school;

x_2_-13 yr of school; x_3_-18 yr of school (university).

All subjects gave written informed consent to the procedures approved by the Institutional Human Subjects Ethics Committee (Heinrich-Heine-University Duesseldorf).

### Design and instruments

**Figure 1:**
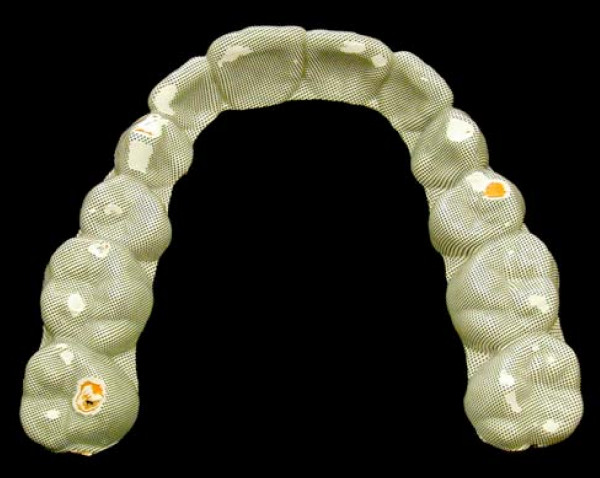
**The BBMD, worn by a SB-patient for five consecutive nights, showing all abraded layers of the plate**.

**Figure 2:**
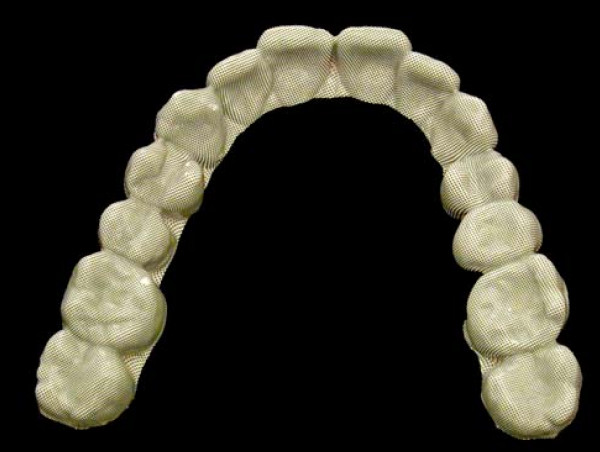
**The BBMD, worn by a non-bruxing subject for five consecutive nights**.

In order to quantify current SB its degree was measured using the BBMD. The BBMD is a 0.51 mm thick plate, consisting of four laminated polyvinyl chloride sheets of different colors and a halftone dot screen on the topmost surface (Figure 1 and 2). The BBMD combined with a semi-automatic computer-based analyzing method has been investigated in a previous study and is described as a clinically feasible and reliable instrument with good sensitivity and specificity parameters, which allows quantification of current SB over a short period [45]. The BBMD had to be worn for five consecutive nights in the upper jaw. The computer-based analysis for quantification of abrasion on the BBMD was done analogue to the procedure described by *Ommerborn et al. *[45] and lead to a so called pixel score.

For the assessment of current stress, subjects completed the following three established and valid German stress- and coping questionnaires: Short questionnaire for recognition of stress-factors (Kurzer Fragebogen zur Erfassung von Belastungen, KFB) [46], Questionnaire for recuperation and strain (Erholungs-Belastungs-Fragebogen, EBF-24 A/3) [47] and the Stress-coping questionnaire (Stressverarbeitungsfragebogen-78, SVF-78) [48]. Following assessment for exclusion criteria and dental examination but prior to the insertion of the BBMD, participants completed the three questionnaires sitting in a quiet room.

The KFB is the short version of a German questionnaire concerning daily life and stress situations [46]. It consists of 16 items, summarized in 4 subscales: 1. 'partnership' (7 items), 2. 'daily problems' (3 items), 3. 'social contacts' (3 items), 4. 'trouble at work' (3 items). Subjects have to quantify their accordance to the several statements on a six-point scale with a range from '0' ('it does not apply') to '5' ('it applies exactly'). Outcomes of the addition of the items form the subscale-values. The total-score of the KFB is calculated by addition of the four subscale-values. The KFB was found to have a satisfying validity [46]. High values for the four subscales imply a high degree of stress in different situations.

The EBF-24 A/3 is the short version of a German questionnaire for the evaluation of a current 'recuperation-strain-balance' [47]. It consists of 24 items, asking for the current balance or imbalance between stressful events and those which help to recuperate. Subjects have to quantify, how often the several events occurred in the last three days with the help of a 7-point response scale. It is suggested that adequate recuperation mechanisms are able to equalize strain. The items are summarized in 12 subscales: 1. 'general strain', 2. 'emotional strain', 3. 'social strain', 4. 'unresolved problems', 5. 'fatigue', 6. 'lack of energy', 7. 'physical problems', 8. 'success', 9. 'social recreation', 10. 'physical relaxation', 11. 'general content', 12. 'sleep'. Subscales 1.-7. are merged to the major scale 'strain' and subscales 8.-12. result in the major scale 'recuperation'. The validity of the questionnaire clearly shows correlations to the current mental state [47]. High values for the scale 'strain' imply a high amount of stressful events, whereas high values for the scale 'recuperation' imply a high level of events that help to recuperate and to equalize strain.

The SVF-78 is the short version of a German coping questionnaire [48]. It consists of 78 items (5-point response scale, 0-4), which survey the different strategies of coping. The basic assumption of the questionnaire is that these strategies are relatively situation-invariant traits, which are thought to be stable over time. The items are summarized in 13 subscales: 1. 'self-aggrandizement by comparison with others', 2. 'denial of guilt', 3. 'distraction', 4. 'substitute gratification', 5. 'situation control', 6. 'reaction control', 7. 'positive self-instructions', 8. 'need for social support', 9. 'avoidance', 10. 'escape', 11. 'rumination', 12. 'resignation', 13. 'self-blame'. Subscales 1. to 8. are merged to the major scale 'positive coping strategies'. They represent strategies, appropriate to reduce stress. Subscales 9 to 13 result in 'negative coping strategies' which tend to enhance stress. The SVF-78 is the most established and most frequently applied coping-questionnaire in Germany [49]. It is the only well evaluated instrument, investigating general coping styles as a stable trait in German language [50].

Validity of the SVF-78 has been proved by factorial analysis and correlation with divergent and convergent traits [48,51].

High values for positive coping-strategies imply a high degree of strategies that are appropriate to reduce stress, whereas high values for negative coping-strategies imply a high degree of strategies that are prone to augment stress.

### Statistical analysis

The statistical analysis was performed using the statistical software 'SPSS' Version 15.0 (SPSS^® ^Software GmbH, Munich). Non-parametric Spearman correlation coefficients, rho, were calculated between the psychometric data and the pixel score of the BBMD for the whole sample of n = 69 subjects. For the detection of mean-differences between the two groups concerning the sociodemographic characteristics and data related to the type of occlusal guidance scheme t-test and Chi-square test were applied (Table 1).

An α-error probability of p < 0.05 was adopted as the statistically significant level.

For the interpretation of the correlations' degree, with reference to the value of the correlation coefficient r, the classification according to *Zöfel *was applied [52].

According to this classification a value between 0.2 and 0.5 indicates a low correlation and a value between 0.5 and 0.7, a moderate correlation.

## Results

### Sociodemographic data

The analysis of the sociodemographic characteristics showed, that the number of female subjects was about two times higher than the number of male subjects. Furthermore SB-patients did not differ significantly from non-bruxing subjects with regards to age, gender and education. In addition, SB-patients and non-bruxing subjects corresponded in terms of occlusal guidance (Table 1).

### Psychometric data and pixel score

Results showed that different subscales of both the stress questionnaires and the coping questionnaire correlate significantly with SB. The most distinct correlation could be seen between the subscale 'daily problems' of the KFB and the pixel score. This demonstrated that the more the problems in daily life, the higher was the degree of SB. For the KFB, it could also be shown, that SB descriptively increased with increasing 'trouble at work', even though the correlation coefficient was lower than for 'daily problems'. In conjunction, these two subscales reveal the significant correlation between the total-score of the KFB and the pixel score. Moreover, significant correlations were found between the pixel score and the subscales 'fatigue' and 'physical problems' of the EBF-24 A/3. Both 'fatigue' and 'physical problems' belong to the major scale 'strain'. The more exhausted the subjects were and the more physical problems they had, the more they ground their teeth. All other variables of the KFB and EBF-24 A/3 did not show any significant correlations (Table 2 and Table 3).

**Table 2:** Spearman correlation coefficient rho and *P*-value for the correlations between the subscales of the KFB and the pixel score of the BBMD (n = 69)

Subscale	*P*	Correlation coefficient
Partnership	n. s.	r = 0.075
Daily problems	***	r = 0.461
Social contacts	n. s.	r = -0.057
Trouble at work	*	r = 0.293

Total Score	**	r = 0.348

n.s. = not significant; * = p < 0.05; ** = p < 0.01; *** = p < 0.001

A positive significant correlation between the factors 'daily problems' and 'trouble at work' with the pixel score was found.

**Table 3:** Spearman correlation coefficient rho and *P*-value for correlations between the subscales of the EBF-24 A/3 and the pixel score of the BBMD (n = 69)

Subscales	*P*	Correlation coefficient
General strains	n. s.	r = 0.022
Emotional strains	n. s.	r = -0.052
Social strains	n. s.	r = 0.107
Unresolved problems	n. s.	r = 0.110
Fatigue	*	r = 0.288
Lack of energy	n. s.	r = 0.127
Physical problems	*	r = 0.288
Success	n. s.	r = -0.087
Social recreation	n. s.	r = 0.023
Physical relaxation	n. s.	r = -0.140
General content	n. s.	r = -0.105
Sleep	n. s.	r = 0.203

n.s. = not significant; * = p < 0.05; ** = p < 0.01; *** = p < 0.001

Positive significant correlations between the factors 'fatigue' and 'physical problems' and the pixel score were found.

Regarding the coping strategies of subjects, the significant correlation found between the pixel score and the subscale 'escape' of the SVF-78 indicated that the more the subjects fled their problems and did not deal with stress in a positive way, the higher was their SB-activity (Table 4).

**Table 4:** Spearman correlation coefficient rho and *P*-value for correlations between the subscales of the SVF-78 and the pixel score of the BBMD (n = 69)

Subscales	*P*	Correlation coefficient
Self-aggrandisement by comparison with others	n. s.	r = -0.046
Denial of guilt	n. s.	r = 0.050
Distraction	n. s.	r = 0.122
Substitute gratification	n. s.	r = 0.030
Situation control	n. s.	r = 0.000
Reaction control	n. s.	r = -0.086
Positive self-instructions	n. s.	r = -0.219
Need for social support	n. s.	r = -0.107
Avoidance	n. s.	r = -0.149
Escape	*	r = 0.295
Rumination	n. s.	r = 0.072
Resignation	n. s.	r = 0.077
Self-blame	n. s.	r = -0.016

n.s. = not significant; * = p < 0.05; ** = p < 0.01; *** = p < 0.001

A positive significant correlation between the factor 'escape' and the pixel score was found.

## Discussion

The present results emphasize the assumption that individuals with high SB-activity seem to feel more stressed in their daily life and at work. This is in accordance with an epidemiological study on British, German, and Italian population samples, where a highly stressful life was positively associated with self-reported SB [21]. Similar results are also provided in a previous study on 1339 employees of a Finnish broadcasting company, which demonstrated that frequent bruxers, regardless of work category, reported more stress. In the same study, frequent bruxism was both significantly positively associated with severe stress experience and with the number of occupational health care and dental visits [22]. In a follow-up study of 30-50 year-old employees (n = 211) of the Finnish broadcasting company it could be reconfirmed that psychosocial factors and perceived stress should not be ignored [17]. The assumption of *Ehlert*, who describes a correlation between stress in daily life ('daily hassle') and the individuals' health status, concerns a similar theory [53]. Similarly, in the present study, it may be assumed that stress could influence the subjects' physical state. This is expressed in the statistically significant values for the subscales ‚fatigue’ and ‚physical problems’ of the EBF-24 A/3. In this context the follow-up study mentioned above could play an important role: it could be shown that, among others, smoking was significantly positively associated with frequent bruxism. It was concluded that tobacco use may both amplify the patient's pain response and provoke bruxism [17]. The factor 'smoking' was however, not controlled for in the present study, so that a possible influence cannot be excluded.

In addition, this study revealed that subjects with high pixel scores do not seem to be able to deal with stress in an adequate way. They seem to prefer negative coping strategies like ‚escape’. This, in general, increases the feeling of stress, instead of looking at the stressor in a positive way. *Schneider et al. *who investigated maladaptive coping strategies in individuals with SB compared to non-bruxing controls observed less positive coping strategies in SB-patients and could therefore also demonstrate a deficit of functional coping strategies for the SB-patients [33].

Similar findings were seen in patients with craniomandibular dysfunction, which demonstrated that these individuals rather daydream, push problems away and do not instruct themselves positively [54]. Other psychosocial factors like trait anxiety [36] coincide with their ineffective reactions like resignation and flight.

The stress questionnaires used in the present study represent valid instruments for the acquisition of stress-parameters of a German population [46-49]. As all subjects were German native speakers, it can be assumed that small semantic differences were also understood.

Due to the fact that all questionnaires were used only for the inquiry of subjective current stress-parameters, meaningful results concerning the correlation between two current parameters - stress and current SB - could be expected. The presence of SB was diagnosed clinically according to the AASM [55] and was done only by one trained dentist, in order to minimize variance. This, however, could be questioned, because clinical criteria like abnormal tooth wear or shiny spots on restorations do not verify the presence of current SB. It could be supposed that the tooth wear had been caused many years ago. Nevertheless, criteria employed in the study, like sleeping partner's report of grinding sounds in combination with the self-report of muscle fatigue or pain on awakening give a clear hint towards current SB-activity. Furthermore, a previous investigation has verified the ability of the BBMD combined with the computer-based analyzing method, used in this investigation, to record current SB-activity [45].

In summary, several stress-parameters described here, concerning specific stress-factors as well as stress-coping strategies correlated significant with SB. Essentially, the stress-factor 'daily problems' seems to play an important role for increasing SB-activity, expressed by a high significance-level and moderate correlation coefficient r, according to the scale of *Zöfel *[52]. Although causal relationships between stress and SB cannot be concluded due to the descriptive character of the statistical analysis, the present results may be interpreted as a promising hint about an existing relationship between stress and SB. However, since, to date, the etiology of SB remains unclear, a moderate correlation between factors like abrasion on a plate, verified as current SB by the computer-based analyzing method, and stress gives enough ground for future investigations in this field in order to identify possible causal relationships between stress, stress-coping and SB.

Concluding from the above, it is worthwhile to further examine stress as one possible etiological factor for the development of SB in experimental trials, preferably in a sleep laboratory. Of interest would be a study designed to measure stress-parameters, stress-reaction and the SB-activity of different subjects, who are exposed to the same stressor, regularly for a longer, defined observation period. Such a study design would allow identification of the different types of daily life events and factors at work that are believed to be stressful. Furthermore, it would be interesting to investigate the reasons due to which individuals in a similar stress situation feel stressed at times and not at others. In order to understand the parameter 'coping' more precisely, further research needs to be carried out into the following: what leads to the development of positive or negative coping strategies?, to what extent does stress influence and modify the type of coping strategies?, and whether positive coping of daily problems reduces SB-activity. In the same context, it should additionally be examined, whether SB itself could lead to stress and/or maladaptive coping strategies as a result of the chronic dental and/or myofascial disturbance and a possible subsequent helplessness felt by the patient.

## Conclusions

Within the limitations of this study it could be shown that subjects with high SB-activity tend to feel more stressed at work and in their daily life, which in turn might influence their physical state. These subjects also seem to deal with stress in a negative way. However, due to the rather low to almost moderate correlation coefficients and the descriptive character of the study, further investigations are necessary to examine a possible causal relationship

## Competing interests

The authors declare that they have no competing interests.

## Authors' contributions

MAO, CS and RS conceived the study design, MG fabricated the BBMDs, performed its analysis as well as the statistical data analysis and wrote the manuscript. CS conducted the psychological part of this study, MAO executed the dental part of this study, PS made the necessary language correction in the manuscript, MF and WHMR participated in the early preparation of the manuscript and contributed to writing the article. All authors have read and approved the final manuscript.
